# Biofertilizers regulate the soil microbial community and enhance *Panax ginseng* yields

**DOI:** 10.1186/s13020-019-0241-1

**Published:** 2019-05-23

**Authors:** Linlin Dong, Yong Li, Jiang Xu, Juan Yang, Guangfei Wei, Liang Shen, Wanlong Ding, Shilin Chen

**Affiliations:** 1 Key Laboratory of Beijing for Identification and Safety Evaluation of Chinese Medicine, Institute of Chinese Materia Medica, China Academy of Chinese Medical Sciences, Beijing, 100700 China; 2 Institute of Medicinal Plant Development, Chinese Academy of Medical Sciences & Peking Union Medical College, Beijing, 100193 China

**Keywords:** *Panax ginseng*, Biofertilizer, Soil microbial community, Root-rot, High-throughput sequencing

## Abstract

**Background:**

*Panax ginseng* is widely used as functional food and traditional Chinese medicine. To satisfy the market supply and medication safety, biofertilizers are used as agents to stimulate the growth and production of *P*. *ginseng*.

**Methods:**

In this study, we used high-throughput sequencing and quantitative polymerase chain reaction to analyze microbial community in soils treated with biofertilizers during the development stages of *P*. *ginseng*. Ginsenoside content was detected using high-performance liquid chromatography analysis to evaluate the effects of biofertilizer application.

**Results:**

In this study, the incidence rate of *P*. *ginseng* root rot significantly declined by 40.3–47.3% after application of disease-biocontrol biofertilizers. Bacterial diversity showed increasing trends in soils treated with 3.0–4.5 ml kg^−1^ of disease-biocontrol biofertilizers compared with those in untreated soils. Principal coordinate analysis ordination revealed that bacterial communities were changed by biofertilizers depending on their application concentration. Relative abundance of potentially beneficial bacterial agents, such as *Bacillus*, *Burkholderia*, *Rhizobium*, *Streptomyces*, and *Mycobacterium*, significantly increased compared with that in control. *Fusarium* of low abundance observed in soils treated with biofertilizers compared with that in untreated soils. *P*. *ginseng* yield was enhanced by 17.0–19.1%, and ginsenoside (Rg1 and Rb1) contents were improved after biofertilizer application.

**Conclusions:**

Our results reveal that biofertilizers reduced the incidence rate of root rot, increased bacterial diversity, promoted the relative abundance of potentially beneficial bacterial taxa, decreased the abundance of potentially harmful bacterial agents, and then enhanced the yield and quality of *P*. *ginseng*.

**Electronic supplementary material:**

The online version of this article (10.1186/s13020-019-0241-1) contains supplementary material, which is available to authorized users.

## Background

*Panax ginseng* is known for its value to human health; it boosts energy, reduces stress, and lowers blood sugar and cholesterol levels [1, 2]. At present, *P*. *ginseng* cultivation is performed to satisfy the market demands. The use of fertilizer is an effective strategy to increase the supply of *P*. *ginseng*. However, the overuse of agrochemicals has resulted in massive ecological degradation, including soil infertility, increased salinity, biodiversity loss, and soil-borne diseases [3, 4]. *Panax* root rot is a severe soil-borne disease that spreads rapidly and causes considerable yield loss [5, 6]. *Fusarium oxysporum* is one of the main pathogens of *Panax* plants [7, 8]. Chemical pesticides are used to control disease, but adsorption of residual pesticides by roots can result in serious health issues [9]. Sustainable *P*. *ginseng* production requires novel pathways to reduce the occurrence of disease and the application of agrochemicals or pesticides that can pollute the environment. Safety biofertilizers and strategies to control soil-borne diseases are urgently needed for the *P. ginseng* cultivation.

Biofertilizers are regarded as environmentally sustainable and cost-effective alternative to synthetic fertilizers [10, 11]. Biofertilizers are products that contain living microorganisms or natural compounds from organisms that regulate soil biological properties, improve plant growth, restore soil fertility, and decrease plant diseases [12, 13]. Extensive reports on biofertilizers have revealed their capacity to provide nutrients to plants and consequently enhance crop yield [14, 15]. Most commercialized rhizobacterial products serve as bioinoculants to antagonize plant diseases [16]. To satisfy market demands and medication safety requirements, biofertilizers are used as agents to stimulate the growth and yield of *P*. *ginseng* by increasing the nutrient supply or inhibiting plant diseases.

Microbes are living soil members that play important roles in soil ecosystem activities that promote nutrient mobilization, plant growth, and yield [17, 18]. Variations in diversity and composition of soil microbial communities disrupt the ecological function and negatively affect soil productivity, thereby leading to yield loss [19]. Regulation of soil microbial communities by inoculating functional microbes can renovate microecology and improve crop yield [20, 21]. Microorganisms also serve as antagonists to soil-borne phytopathogens. Inoculation of *Bacillus subtilis* 50-1, for example, alleviates the replanting mortality of *P. ginseng* [8]. *P. ginseng*, a perennial plant undergoing different developmental stages annually, is continuously cultivated for 4–5 years prior to harvest. While significant shifts in the diversity of soil microbial communities have been observed during the developmental stages of this plant [8]; few reports are available regarding how biofertilizers affect *P. ginseng* yield by regulating soil microbial communities during these stages.

*Panax ginseng* is a traditional Chinese medicine, and its main active components are ginsenosides, including Rg1, Re, Rb1, Rc, Rf, Rd, and Rb2 [22]. Ginsenoside content is a vital quality index in the market. Zhao et al. [23] reported that *B*. *amyloliquefaciens* GB03 inoculation improves metabolite accumulation in *Codonopsis pilosula* (Franch.). We speculate that application of biofertilizers affects the ginsenoside content of *P. ginseng*. In this study, we analyzed changes in soil microbial communities under biofertilizer treatment through high-throughput sequencing analysis of 16S RNA genes during the different developmental stages of *P*. *ginseng.* To evaluate the effects of biofertilizers on potentially harmful taxa, we examined the abundance of *Fusarium* through quantitative polymerase chain reaction (qPCR). Ginsenoside content was detected using high-performance liquid chromatography to evaluate the effects of biofertilizer application. Our results indicated that biofertilizer application could contribute to the sustainable cultivation and the safe medical use of *P*. *ginseng*.

## Materials and methods

### Site description and field experiment design

A field experiment was performed in a *P*. *ginseng* plantation in Jingyu, Jilin Province (42°20ʹN, 126°50ʹE, 775 m a.s.l.). This experimental site was introduced in the description of Dong et al. [8]. The field area is a traditional farmland with corn, and it has served as a cultivation area of *P*. *ginseng* since 2014. This area was subdivided in 21 plots with 1.6 m × 6.0 m. The field experiment comprised seven treatments, including treatment with growth-promoting biofertilizers (T1), treatment with disease-biological biofertilizers (T2), and no biofertilizer treatment (CK). The two types of biofertilizers included three concentrations for three treatments. A total of 3.0, 6.0, and 9.0 ml kg^−1^ of growth-promoting biofertilizers represented low (T1-L), middle (T1-M), and high (T1-H) concentrations, respectively. Furthermore, 1.5, 3.0, and 4.5 ml kg^−1^ of disease-biocontrol biofertilizers represented low (T2-L), middle (T2-M), and high (T2-H) concentrations, respectively. T1 of *P*. *ginseng* comprised a liquid gel suspension containing a phototrophic and N-fixing bacteria consort (*Burkholderia* and *Rhizobium*) cultured in vitro. The gel contained over 8000 propagules per milliliter. T2 of *P*. *ginseng* contained a liquid gel suspension with Gram-positive actinomycetes (*Actinomyces*), *Bacillus* and *Aspergillus*. The gel comprised over 10,000 propagules per milliliter. Different biofertilizer concentrations with organic fertilizer were uniformly mixed and fermented for 30 days with polyethylene film. The main component of the organic fertilizer was pig and cow manure (W/W = 1:2), which was provided by Shengshi Baicao, China. Each biofertilizer concentration with organic fertilizers (3.0 kg m^−2^) was randomly applied to one plot as one treatment. Moreover, the application of organic fertilizers without biofertilizers served as the control. This experiment was carried out in triplicate. After 1 month of application, 2-year-old ginseng seedlings (15 cm × 10 cm) were transplanted into each plot (1.6 m × 6.0 m) in 2014. *P*. *ginseng* was cultivated according to the standard operating procedures of good agricultural practice [24, 25]. *P*. *ginseng* seedlings are commonly cultivated for 4–5 years before harvest, and they undergo the different developmental stages of vegetative, flower, fruit, and root growth after transplantation. To determine the effectiveness of the biofertilizers applied, we analyzed the growth and soil microbial community during the different developmental stages of *P*. *ginseng* and assessed the yield and quality of 4-year-old seedlings in 2016.

### The incidence rate of *P. ginseng* root rot

Root rot is a serious *P*. *ginseng* disease that occurs from June to August each year. *P*. *ginseng* root rot was investigated from June to August, and the incidence rate was calculated in August 2015 and 2016 after transplantation. The incidence rate was calculated as the number of diseased plants divided by the number of all plants in each plot (1.6 m × 6.0 m). This experiment was carried out in triplicate.

### Soil microbial communities

To analyze changes in the soil microbial community under biofertilizer treatment, we collected soil samples from different developmental stages of *P*. *ginseng* seedlings. The sampling times of the developmental stages of vegetative, flower, fruit, and root growth corresponded to the description of Dong et al. [8]. Soil samples were collected from five spots (0–20 cm) around each seedling root by the drilling method. Soil samples from six seedlings randomly selected from each plot were pooled into one sample. Three replicate were prepared. All samples were homogenized by passing through a 2 mm sieve for analysis of soil microbial communities. Total soil DNA was extracted using a MoBio Powersoil Kit (MoBio Laboratories Inc., Carlsbad, CA) in accordance with the usage guidelines. DNA samples were stored at − 20 °C for further processing. Fragments of 16S rRNA genes were amplified using the primers 27F/338R [26]. These primers were with an 8 bp pair barcode to analyze the soil microbial communities (Additional file 1: Table S1). Amplification, purification, and quantitation were performed according to the description of Rodrigues et al. [27]. The pooled DNA products were used to construct an Illumina pair-end library and subsequently pair-end sequenced (2 × 250) on an Illumina HiSeq platform based on standard protocols.

Data were quality filtered using QIIME according to the standard pipeline [28]. Operational taxonomic units (OTUs) were clustered using a 97% similarity cut-off via UPARSE version 7.1 (http://drive5.com/uparse/). Chimeric sequences were identified using UCHIME. The phylogenetic affiliation of 16S rRNA gene sequences was analyzed by the Ribosomal Database Project [29]. Rarefaction analysis according to Mothur v.1.21.1 was performed to determine the diversity indices, including ACE, Chao 1, and Shannon diversity indices. The taxa obtained from RDP Classifier through complete linkage hierarchies were clustered using *R* package HCLUST (http://sekhon.berkeley.edu/stats/html/hclust.html). Principal coordinate analysis (PCoA) analysis was performed to compare groups of samples according to Bray–Curtis distance metrics. All data from high-throughput sequencing were submitted to the National Center for Biotechnology Information (http://www.nvbi.nlm.nih.gov), and the accession number of the 16S rRNA sequences was SRP131253.

To evaluate the effects of biofertilizers on potential pathogenic agents, we analyzed the abundance of *Fusarium* by quantitative PCR analysis. *Fusarium* fragments were amplified using the primers ITS-Fu-F/ITS-Fu-R [30]. The copy numbers of *Fusarium* in soils of *P*. *ginseng* seedlings among the different treatments were calculated according to the previous description [8, 31].

### The root growth and yield of *P. ginseng*

To evaluate the effects of biofertilizers, the fresh root weight and yield of 4-year-old *P*. *ginseng* seedlings were analyzed in October 2016. A total of 30 plants were used to analyze root growth [32]. Ten *P*. *ginseng* seedlings were randomly collected from each plot to analyze fresh root weight. *P*. *ginseng* seedling roots randomly collected from 1 m^2^ of each plot under different treatments were dried to calculate yields [33]. Data reflect the mean of triplicates.

### Ginsenoside contents in *P. ginseng* root

The standard ginsenosides, namely, Rg1, Re, Rb1, Rb2, Rc, and Rd, were purchased from Shanghai Tauto Biotech Company (Shanghai, China). The standard solutions were dissolved in methanol (Fair Lawn, NJ, USA) for further experiments. Ten samples of *P. ginseng* roots (4-year-old) from each plot were pooled and considered one sample. *P*. *ginseng* extract preparation was performed according to the description of Dong et al. [22]. Ginsenoside contents were determined through high-performance liquid chromatography (Agilent 1260, USA) using a system equipped with a binary pump, an online degasser, a column compartment, and an auto plate sampler. The reA C18 reversed phase column (250 mm × 4.6 mm, i.d. 5 µm; Eclipse XDB, Agilent, USA) was used for separation. The column temperature was kept at 25 °C, and the flow rate and wavelength were 1.0 ml min^−1^ and 203 nm, respectively. The gradient was composed of water and acetonitrile. The linear gradient was set as follows: 0–12 min for 19% acetonitrile and 12–60 min for 19–40% acetonitrile.

### Statistical analysis

Variables were used to analyze replicates and subjected to ANOVA using SPSS version 16.0 software (SPSS Inc., Chicago, IL, USA). The data were presented as mean ± SD of n = 3. No adjustments were implemented for multiple comparisons. Duncan’s multiple range test was used to analyze differences between means at a significance level of *P *< 0.05.

The Minimum Standards of Reporting Checklist (Additional file 2) contains details of the experimental design, and statistics, and resources used in this study.

## Results

### The incidence rate of root rot decreased after application of disease-biocontrol biofertilizers

**Fig. 1:**
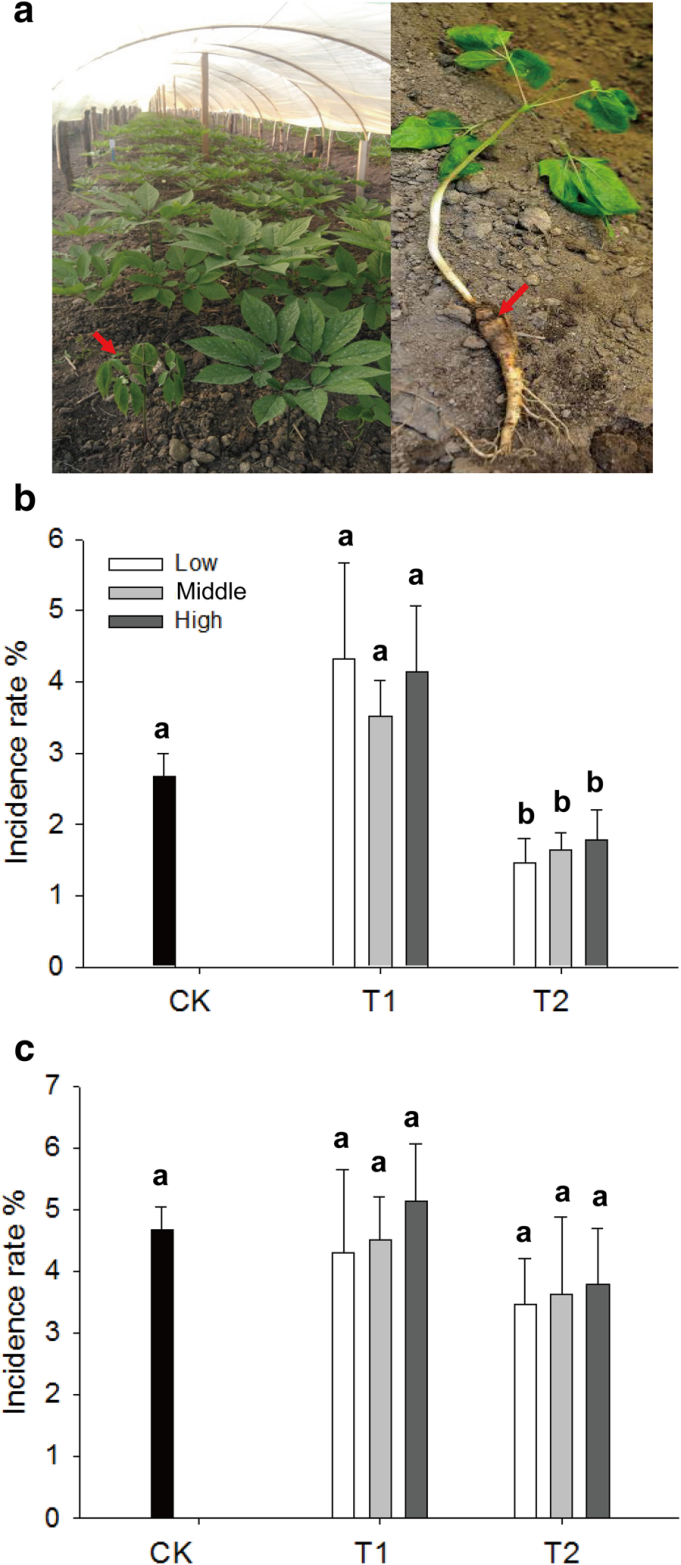
Incidence rate of *Panax ginseng* root rot. **a** Symptoms of root rot. **b** Incidence rate of root rot in 2015. **c** Incidence rate of root rot in 2016. CK represents the treatment without biofertilizers. T1 and T2 represent treatments with growth-promoting and disease-biocontrol biofertilizers, respectively. Data are presented as mean ± SD (*n *= 3). Identical letters denote insignificant differences among treatments at the 0.05 level

The chemical properties (pH, total N, organic content, available P and available K) of soils treated with biofertilizers showed insignificant differences compared with those of untreated soils before *P*. *ginseng* cultivation in 2015 (Additional file 1: Table S2). Insignificant differences in emergence rate and the aboveground growth of *P*. *ginseng* seedlings were also observed in treatments with biofertilizers compared with those in CK (Additional file 1: Figure S1 and Table S3). Root rot infection caused discoloration in *P*. *ginseng* roots and withered aboveground parts (Fig. 1a). The biocontrol effects of T1 on root rot were insignificant compared with those without biofertilizers in 2015 and 2016 (Fig. 1b, c). The influence of T1 on damping-off was insignificant compared with those treatments without biofertilizers in 2015 and 2016 (Additional file 1: Figure S2). The incidence rates of root rot and damping-off significantly decreased by 40.3–47.3% and 30.0–39.4% in T2 compared with those in CK in 2015 (Fig. 1b and Additional file 1: Figure S2). The biocontrol effect of T2 on root rot and damping-off were insignificant compared with those of soils without biofertilizers in 2016 (Fig. 1c and Additional file 1: Figure S2).

### Biofertilizers changed the diversity of the soil microbial community

To assess the effects of biofertilizers on soil microecology, we analyzed changes in the soil microbial community in the different developmental stages of *P*. *ginseng* after biofertilizers were applied for 1 year. A total of 6,328,913 classifiable 16S rRNA sequences and 233,542 OTUs were acquired from 84 soil samples for analysis (Additional file 1: Table S1). The mean numbers of sequences and OTUs per sample were 75,344 and 2780, respectively. The OTUs ranged from 2394 to 3122. Compared with that without biofertilizers, the effects of biofertilizers on the bacterial diversity indexes (ACE, Chao, and Shannon) depended on their concentration during the different growth stages of *P*. *ginseng* seedlings (Table 1). Bacterial diversity indices showed increasing trends in T1-M soils (6.0 ml kg^−1^) from the vegetative stage to the root growth stage of *P*. *ginseng* compared with those in untreated soils (CK). Diversity indices also displayed higher values in T2-M and T2-H soils (3.0 and 4.5 ml kg^−1^, respectively) than those in untreated soils from the vegetative stage to the root growth stage of *P*. *ginseng*. In addition, diversity indices were significantly higher in T2-M soils during the vegetative and fruiting stages than in untreated soils. These results indicate that biofertilizers change the diversity of the soil microbial community and that the effects of the biofertilizer depended on the application concentration.

**Table 1:** Alpha diversity of bacterial community in soils with or without biofertilizers

Treatments	Vegetative stage			Flowering stage			Fruiting stage			Root growth stage		
	ACE	Chao	Shannon	ACE	Chao	Shannon	ACE	Chao	Shannon	ACE	Chao	Shannon
CK	5322 ± 298b	4575 ± 91b	6.88 ± 0.05b	5560 ± 275a	4430 ± 188b	6.88 ± 0.08b	5166 ± 184b	4084 ± 138b	6.70 ± 0.03b	5568 ± 134a	4437 ± 153ab	6.91 ± 0.04a
T1-L	5675 ± 452b	4508 ± 56b	6.90 ± 0.07b	5541 ± 148a	4432 ± 100b	6.86 ± 0.06b	5555 ± 354ab	4476 ± 242ab	6.81 ± 0.05b	5505 ± 247a	4476 ± 191ab	6.92 ± 0.02a
T1-M	5491 ± 219b	4597 ± 155b	6.94 ± 0.07b	5624 ± 193a	4480 ± 172b	6.91 ± 0.05ab	5348 ± 193ab	4266 ± 148ab	6.81 ± 0.07b	5724 ± 98a	4698 ± 65ab	7.00 ± 0.03a
T1-H	5637 ± 169b	4529 ± 154b	6.91 ± 0.08b	5589 ± 232a	4462 ± 158b	6.86 ± 0.07b	5467 ± 242ab	4342 ± 198ab	6.81 ± 0.07b	5315 ± 143b	4277 ± 83b	6.86 ± 0.03b
T2-L	6168 ± 57a	4930 ± 33a	7.09 ± 0.02a	5875 ± 113a	4654 ± 119a	6.95 ± 0.06a	4966 ± 268b	4293 ± 59ab	6.81 ± 0.08b	5622 ± 86a	4516 ± 99ab	6.96 ± 0.05a
T2-M	6127 ± 89a	4940 ± 52a	7.07 ± 0.04a	5938 ± 55a	4783 ± 34a	7.00 ± 0.02a	5788 ± 181a	4613 ± 120a	6.95 ± 0.05a	5824 ± 68a	4726 ± 16a	7.00 ± 0.01a
T2-H	5870 ± 136b	4749 ± 46b	6.98 ± 0.02b	5836 ± 186a	4586 ± 312b	6.90 ± 0.04ab	5332 ± 124ab	4339 ± 134ab	6.80 ± 0.09b	5667 ± 143a	4515 ± 90ab	6.94 ± 0.05a

CK represents the treatment without biofertilizers. T1-L, T1-M and T1-H present treatments with growth-promoting biofertilizers at low, middle, and high concentrations, respectively. T2-L, T2-M, and T2-H present treatments with disease-biocontrol biofertilizers at low, middle, and high concentrations, respectively. Data are presented as mean (*n *= 3) ± SD. Identical letters denote non-significant difference at 0.05 level

### Biofertilizers changed the soil microbial community

**Fig. 2:**
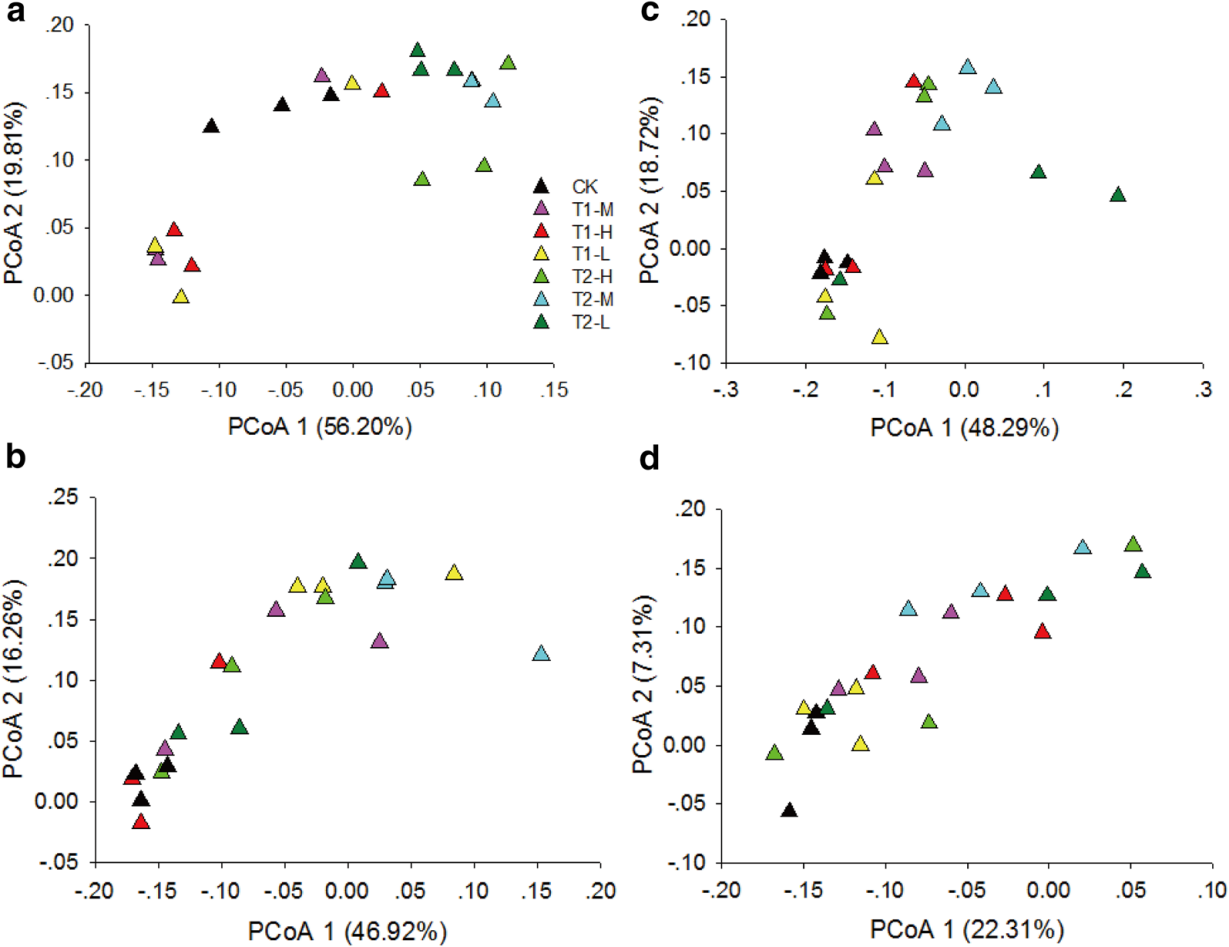
Changes in bacterial communities in soils with and without biofertilizer. **a**–**d** Principal coordinate analysis ordination plots show the relatedness of samples separated using the Bray–Curtis distances of the classified 16S rRNA gene sequences at the vegetative, flowering, fruiting, and root growth stages. CK represents the treatment without biofertilizers. T1-L, T1-M, and T1-H represent treatments with growth-promoting biofertilizers at low, middle, and high concentrations, respectively. T2-L, T2-M, and T2-H represent treatments with disease-biocontrol biofertilizers at low, middle, and high concentrations, respectively

PCoA ordination revealed changes in the bacterial communities in T1 and T2 soils compared with that in CK soils (Fig. 2). During the vegetative stage of *P*. *ginseng*, the first principal component (56.20% contribution) demonstrated bacterial communities in T2-L, T2-M, and T2-H soils compared with those in CK (Fig. 2a). During the flowering stage of *P. ginseng*, the first principal component axis (46.92% contribution) showed that bacterial communities in T2-M soils markedly differed from those in T1-H, T2-H, and CK soils (Fig. 2b). During the fruiting stage of *P*. *ginseng*, the second principal component axis (18.72% contribution) indicated that the bacterial communities in T1-M and T2-M soils differed from those in CK (Fig. 2c). During the root growth stage of *P*. *ginseng*, PCoA ordination showed insignificant differences in bacterial communities between soils treated with biofertilizers and CK (Fig. 2d).

### Biofertilizers changed the relative abundance of bacterial groups

**Fig. 3:**
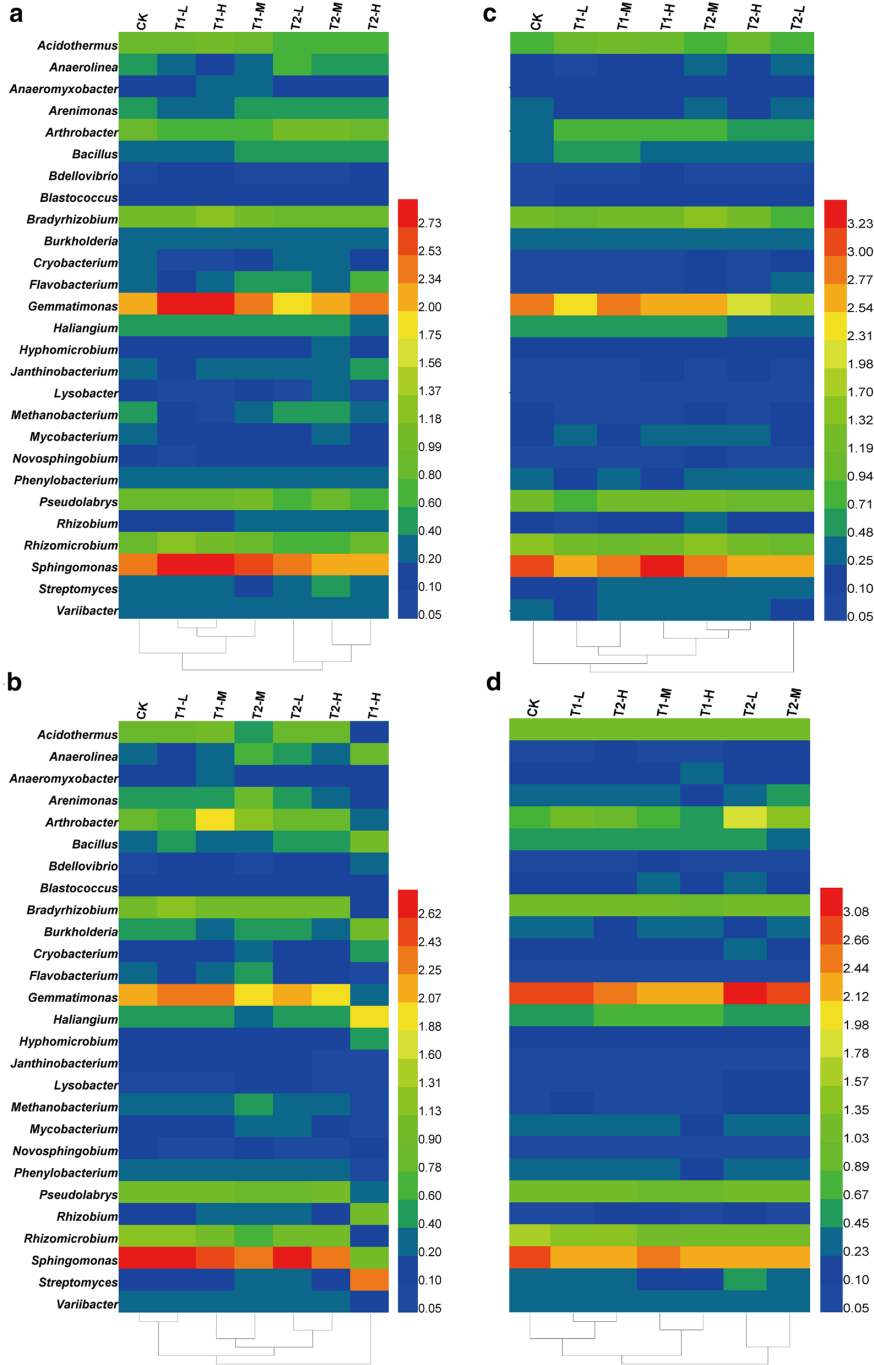
Changes in bacterial genera in soils with and without biofertilizers. **a**–**d** Changes in the relative abundance of bacterial genera at the vegetative, flowering, fruiting, and root growth stages. Heat map showing major bacterial genera with average relative abundance > 0.05% in all samples. Clustering on the x-axis relied on the bacterial composition of the samples. CK represents the treatment without biofertilizers. T1-L, T1-M, and T1-H represent treatments with growth-promoting biofertilizers at low, middle, and high concentrations, respectively. T2-L, T2-M, and T2-H represent treatments with disease biocontrol biofertilizers at low, middle, and high concentrations, respectively. Data are presented as mean (*n *= 3)

The relative abundance of bacterial groups changed in treated or untreated soils from the phylum level to the genus level during the different developmental stages of *P*. *ginseng* (Additional file 1: Figures S3, S4 and Fig. 3). *Acidobacteria*, *Actinobacteria*, *Chloroflexi*, *Gemmatimonadetes*, *Proteobacteria*, and *Verrucomicrobia* were the main bacterial taxa at the phylum level in soils of *P*. *ginseng* (Additional file 1: Figure S3). During the flowering and fruiting stages of *P*. *ginseng*, the relative abundance of *Gemmatimonadetes* showed decreasing trends in T1 and T2 soils compared with those in CK soils. Additionally, *Chloroflexi* abundance increased in T1 and T2 soils compared with those in CK soils during the flowering, fruiting, and root growth stages of *P*. *ginseng*. The relative abundance of bacterial groups showed fluctuations at the family level in treated or untreated soils (Additional file 1: Figure S4).

During the vegetative stage of *P*. *ginseng*, the relative abundances of *Anaerolinea*, *Cryobacterium*, and *Methanobacterium* significantly declined by 54.5–69.5%, 24.8–28.2%, and 48.9–88.5%, respectively, in soils with biofertilizers compared with those in soils without biofertilizers. The relative abundances of *Anaeromyxobacter*, *Bdellovibrio*, and *Sphingomonas* increased by 56.0–82.3%, 11.4–65.7%, and 17.6–21.6%, respectively, in T1 soils compared with those in CK soils (Fig. 3a). Moreover, the relative abundance of *Bacillus*, *Flavobacterium*, *Rhizobium*, and *Streptomyces* increased by 30.3–57.2%, 10.2–82.5%, 77.9–116.7%, and 14.9–102.0%, respectively. The relative abundances of *Haliangium* and *Methanobacterium* decreased by 10.8–17.1% and 15.4–33.4%, respectively, in T2 soils compared with those in CK soils.

During the flowering stage of *P. ginseng*, the relative abundances of *Arenimonas* and *Pseudolabrys* declined by 12.0–64.0% and 12.0–78.7%, respectively, in soils with biofertilizers compared with those in soils without biofertilizers. The relative abundance of *Bdellovibrio* markedly increased by 37.8–303.4% in T1 soils compared with those in CK soils (Fig. 3b). The relative abundances of *Bacillus*, *Burkholderia*, *Rhizobium*, and *Streptomyces* significantly increased by 185.7%, 121.1%, 439.0%, and 116.2%, respectively, in T1-H soils compared with those in CK soils. The relative abundances of *Anaerolinea* and *Rhizobium* increased by 17.1–230.0% and 14.1–125.6%, respectively, in T2 soils compared with those in CK soils. The relative abundances of *Mycobacterium* and *Streptomyces* markedly increased by 45.0–57.9% and 161.6–179.2%, respectively, in T2-L and T2-M soils compared with those in CK soils.

In the fruiting stage of *P*. *ginseng*, the relative abundances of *Mycobacterium* and *Streptomyces* significantly increased by 11.5–140.0% and 20.3–144.3%, respectively, in T1 and T2 soils compared with those in CK soils (Fig. 3c). The relative abundances of *Acidothermus* and *Bacillus* increased by 37.0–67.2% and 13.3–31.9%, respectively, in T1 soils compared with that in CK soils.

In the root growth stage of *P*. *ginseng*, the relative abundances of *Bacillus*, *Burkholderia*, and *Rhizobium* significantly increased by 28.0%, 28.6%, and 45.3%, respectively, in T1-M soils compared with those in CK soils (Fig. 3d). The relative abundances of *Anaerolinea*, *Arthrobacter*, *Cryobacterium*, and *Janthinobacterium* were remarkably higher in T2 soils than in CK soils. The relative abundances of *Bacillus* and *Rhizobium* significantly increased by 34.3% and 35.3% in T2-L soils compared with those in CK soils.

### Biofertilizers reduced the relative abundance of *Fusarium*

**Fig. 4:**
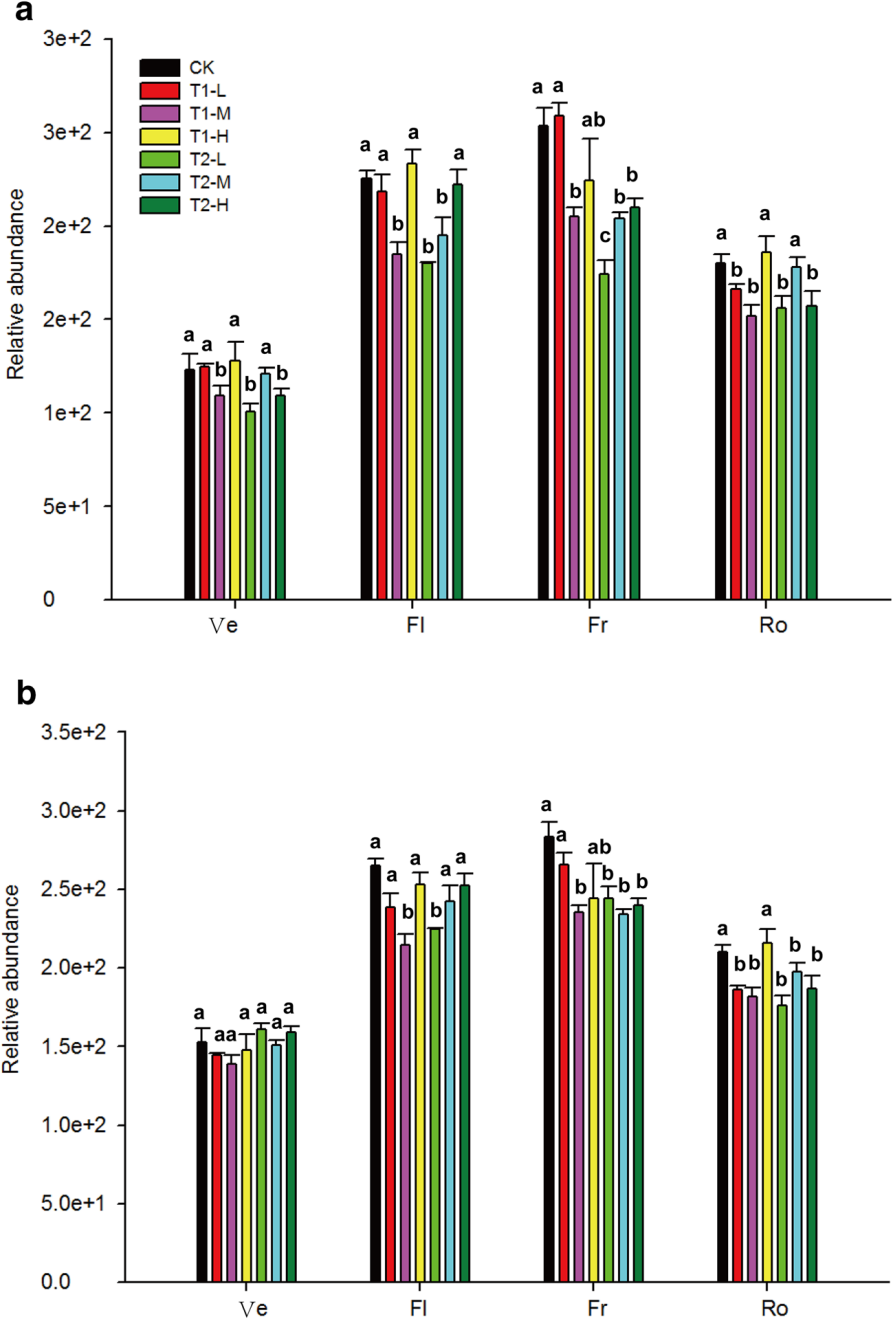
Changes in the relative abundance of *Fusarium* in soils with and without biofertilizers during the different developmental stages of *P*. *ginseng* in 2015 (**a**) and 2016 (**b**). Ve, Fl, Fr, and Ro represent the vegetative, flowering, fruiting, and root growth stages, respectively. CK represents the treatment without biofertilizers. T1-L, T1-M, and T1-H represent treatments with growth-promoting biofertilizers at low, middle, and high concentrations, respectively. T2-L, T2-M, and T2-H present treatments with disease-biocontrol biofertilizers at low, middle, and high concentrations, respectively. Data are presented as mean ± SD (*n *= 3). Identical letters denote insignificant differences among treatments in the same developmental stage at the 0.05 level

The relative abundance of *Fusarium* was reduced in T1 and T2 soils compared with that in CK soils (Fig. 4). The relative abundance of *Fusarium* decreased by 11.4–18.8% and 13.1–31.2% in T1 (middle concentration) and T2 (low concentration) soils, respectively, compared with those in CK soils in 2015 (Fig. 4a). The relative abundance of *Fusarium* declined by 15.9–18.9% in T1 soils (middle concentration) during the flowering, fruiting, and root growth stages of *P*. *ginseng* compared with those in untreated soils in 2016 (Fig. 4b). *Fusarium* abundance significantly decreased in T2 soils during the fruiting and root growth stages of *P*. *ginseng* compared with those in untreated soils in 2016.

### Biofertilizers enhanced the root growth and yield of *P. ginseng*

**Fig. 5:**
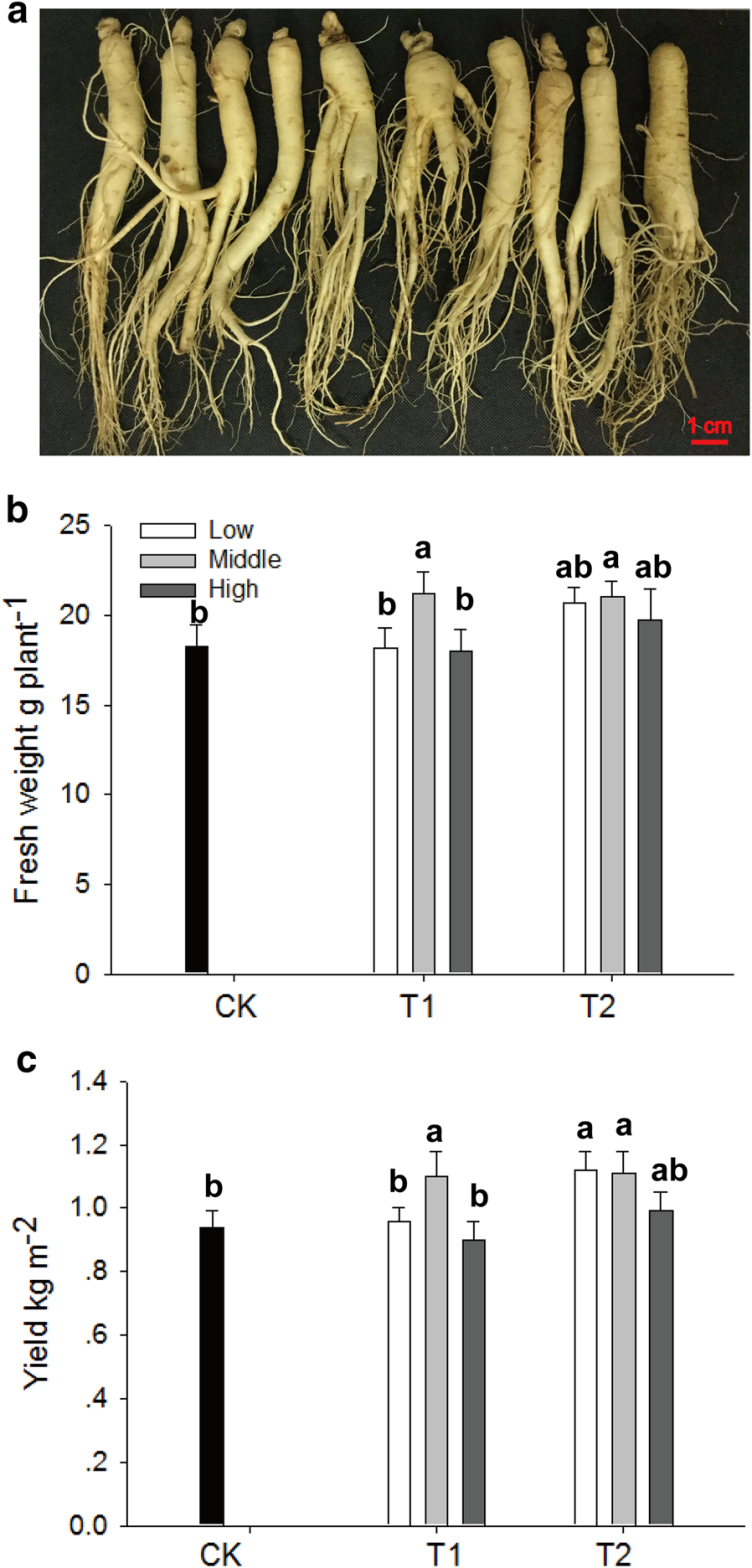
Root growth and yield of *P*. *ginseng*. **a** The 4-year-old *P*. *ginseng* root. **b** Fresh weights of roots in treatments with and without biofertilizers. **c** Yields in treatments with and without biofertilizers. CK represents the treatment without biofertilizers. T1 and T2 represent treatments with growth-promoting and disease-biocontrol biofertilizers, respectively. Data are presented as mean ± SD (*n *= 3). Identical letters denote insignificant differences among treatments at the 0.05 level

The root growth and yield of *P. ginseng* increased depending on the concentrations of biofertilizer application (Fig. 5). While T1-M enhanced the root growth and yield of *P*. *ginseng* by 16.5% and 17.0%, respectively, T2-L and T2-M improved the root growth and yield of the plant by 13.5–15.7% and 18.1–19.1%, respectively.

### Biofertilizers improved the ginsenoside content of *P. ginseng* root

**Fig. 6:**
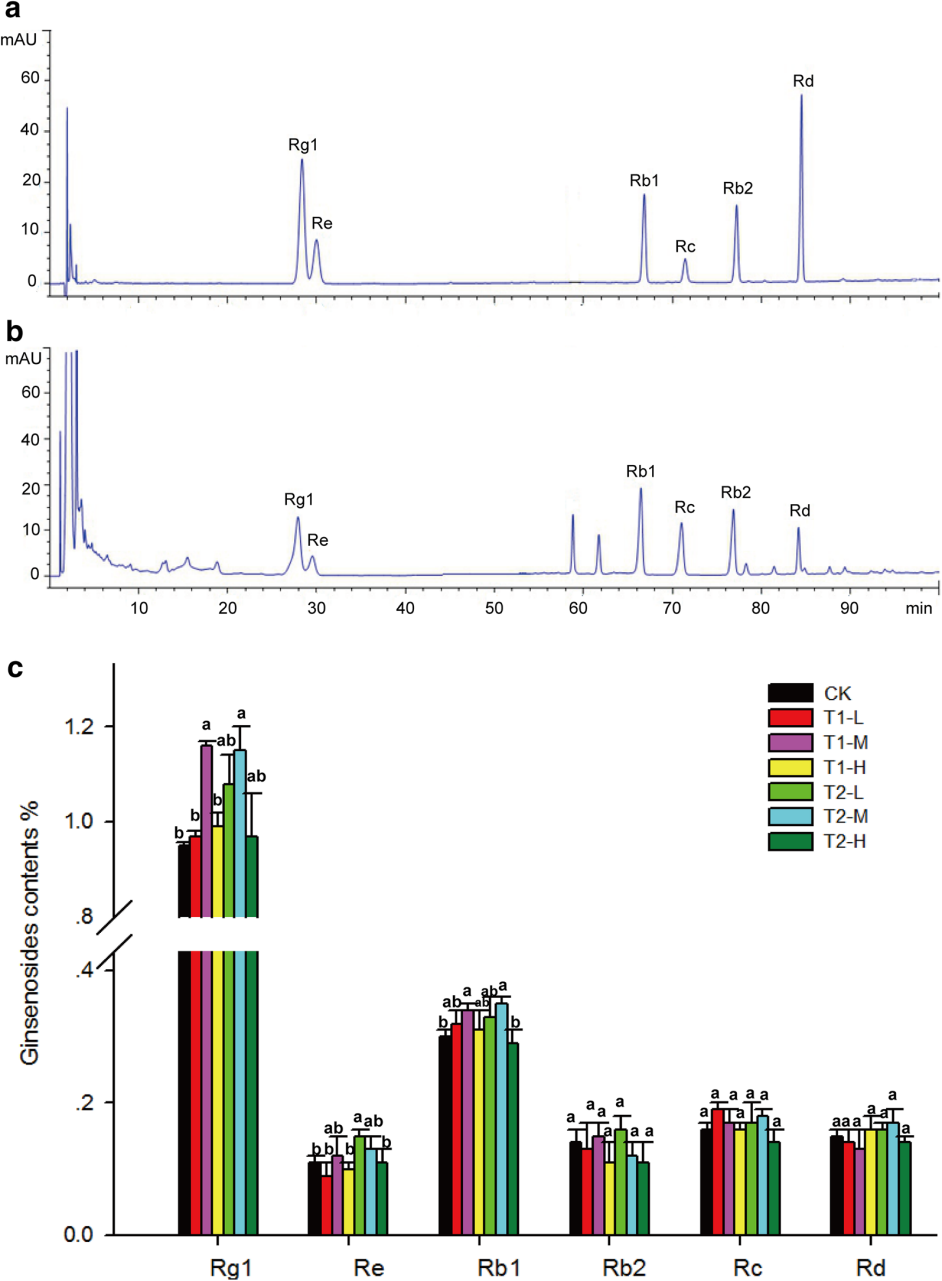
Ginsenoside contents (%) of four-year-old *P*. *ginseng* roots. **a** Mixed standards. **b** HPLC chromatogram profiles of *P*. *ginseng* roots. **c** Contents of Rg1, Re, Rb1, Rc, Rb2, and Rd in *P*. *ginseng* roots. CK represents the treatment without biofertilizers. T1-L, T1-M, and T1-H represent treatments with growth-promoting biofertilizers at low, middle, and high concentrations, respectively. T2-L, T2-M, and T2-H represent treatments with disease-biocontrol biofertilizers at low, middle, and high concentrations, respectively. Data are presented as mean ± SD (*n *= 3). Identical letters denote insignificant differences among treatments at the same developmental stage at the 0.05 level

The ginsenoside (Rg1 and Rb1) content in 4-year-old *P*. *ginseng* roots improved depending on the applied concentration of biofertilizers (Fig. 6). T1-M and T2-M significantly increased Rg1 and Rb1 contents, respectively, compared to those without biofertilizers. The field experimental results indicate that biofertilizers improve the quality of *P*. *ginseng* in a concentration-dependent manner.

## Discussion

The incidence rate of *P*. *ginseng* root rot significantly decreased by 40.3–47.3% through T2 application within 1 year. *P. ginseng* yields and ginsenoside (Rg1 and Rb1) contents were enhanced in T1 and T2 in a concentration-dependent manner. Biofertilizers are widely accepted because they promote plant root growth and protect roots from soil-borne pathogens [34]. The results of biofertilizer application may offer information about the development of disease control strategies [35]. Biofertilizers containing *B. amyloliquefaciens* strain NJN-6 with organic mixtures of pig manure and amino acids can suppress banana *Fusarium* wilt disease [36]. El-Haddad et al. [37] reported that biofertilizers, including phosphate-solubilizing bacteria, that is, *B. circulans*, and N-fixing bacteria, show significant nematicidal activity. In addition, the incidence rate of *P*. *ginseng* damping-off significantly decreased by 30.0–39.4% in T2 within 1 year (Additional file 1: Figure S2). While the incidence rate of *P*. *ginseng* root rot showed increasing trends in T1, these increases were insignificant. Multiple microbial interactions include antibiosis, competition, and symbiosis [38]. Application of microbial inoculants can influence resident microbial communities and disrupt the ecological balance in soil microbial communities [39]. Inoculation of growth-promoting biofertilizers containing living strains can cause imbalance in the rhizosphere and result in the increased incidence rate of root rot. Thus, the biofertilizer type should be considered in practical applications. Bacterial populations enhance yield and serve as antagonists of soil-borne disease [15]. Application of inoculants, such as *B*. *amyloliquefaciens* GB03, improves the metabolite accumulation of *C*. *pilosula* (Franch.) [23]. Li and Huang have reported that application of biological fertilizer improve the indexes of *P. ginseng* growth and total ginsenosides [40]. The yield of *P. ginseng* is increased by 10–30% after application of biofertilizers [41]. Similar to the present results, these findings reveal that biofertilizers can enhance the quality of medicinal materials.

Bacterial diversity indexes evidently increased, and the compositions of soil microbial communities changed in T1 and T2 soils compared with those in CK soils; the effects of biofertilizers depend on their concentrations of application. Biofertilizer application increases the bacterial diversity in soils of cucumber and tobacco [42, 43]. Moreover, biofertilizer application (2 years continuously) regulates the compositions of microbial communities by increasing the bacterial diversity [44]. Dong et al. [45] have reported that biofertilizer application changed the diversity and composition of microbial communities. These results indicated that biofertilizer application manipulated the diversity and compositions of soil microbial communities.

**Fig. 7:**
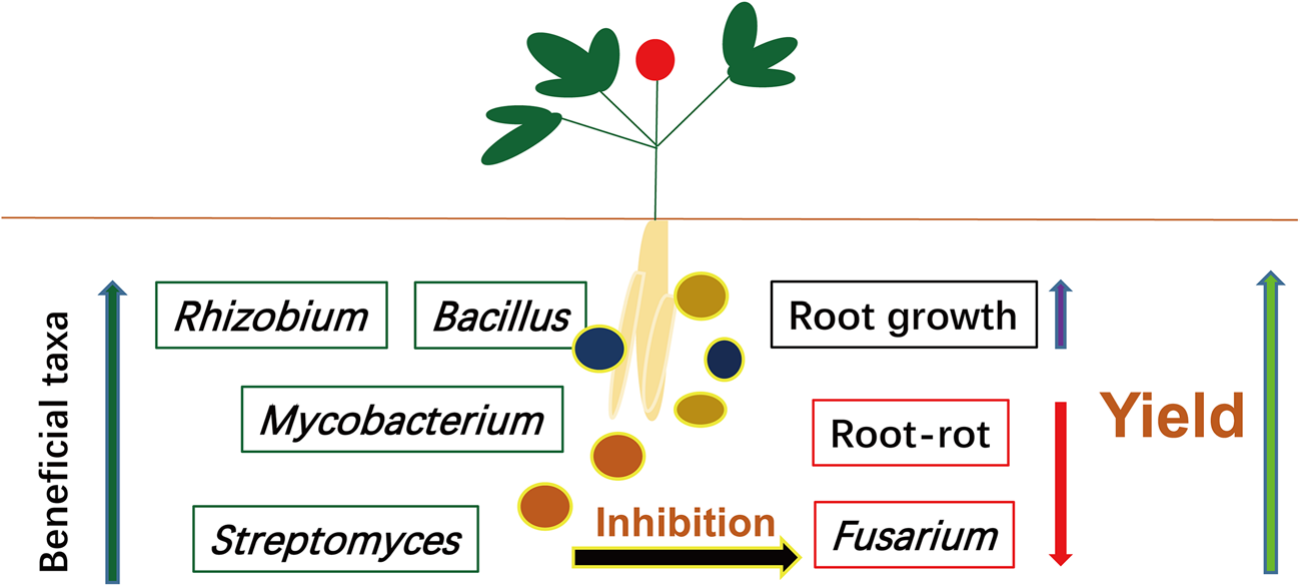
Potential mechanism of biofertilizers regulating the soil microecology to enhance yield. Circles represent bacterial secretions

Plant growth-promoting rhizobacteria (PGPR) directly or indirectly play vital roles in plant health and soil fertility [15]. A large number of PGPR genera, including *Bacillus*, *Enterobacter*, *Burkholderia*, *Pseudomonas*, *Rhizobium*, *Streptomyces*, *Sphingobacterium*, and *Mycobacterium*, have been characterized [46–48]. Evident strains include *Rhizobium*, *Burkholderia*, *Pseudomonas*, and *Bacillus*, which can solubilize insoluble inorganic phosphorus [13]. Many types of biofertilizers, such as *Enterobacter*, *Mycobacterium*, and *Streptomyces*, produce IAA, which induces plant stress resistance [49–51]. Several biofertilizers, such as *Pseudomonas*, produce antifungal antibiotics that can inhibit plant pathological fungi [52]. The abundance of beneficial microbial taxa that can control *Fusarium* wilt disease, such as *Pseudomonas* and *Bacillus*, increased in T1 and T2 soils compared with those in CK soils [44]. In the present study, the biofertilizers contained PGPR, such as a phototrophic and N-fixing bacteria consort, Gram-positive actinomycetes, *Bacillus* and *Aspergillus*. After biofertilizer application, the relative abundance of potentially beneficial bacterial taxa, such as *Bacillus*, *Burkholderia*, *Rhizobium*, *Streptomyces*, and *Mycobacterium*, showed increasing trends. Furthermore, the abundance of *Fusarium* decreased in T1 and T2 soils. The direct mechanism of PGPR involves improvements in N fixation, phosphate solubilization, and phytohormone while its indirect mechanism involves improvements in hydrolytic enzyme production, exopolysaccharide production, and induced system resistance [13]. Taken together, we speculate that application of biofertilizers, including functional groups, promotes *P*. *ginseng* yield by modifying the soil microecology and inhibiting plant pathogenic diseases (Fig. 7). Several beneficial bacterial groups can solubilize phosphate to promote the growth of *P*. *ginseng* root. In addition, other groups secreting antifungal antibiotics can decrease the risk of soil-borne diseases. Our results provided important information about biofertilizer application and contributed to the safe medical use of *P*. *ginseng.*

## Conclusions

In this study, biofertilizers reduced the incidence rate of root rot, increased bacterial diversity, promoted the relative abundance of potentially beneficial bacterial taxa, decreased the abundance of potentially harmful bacterial agents, and enhanced the yield and quality of *P*. *ginseng*. The influence of the biofertilizers depended on their application concentrations. These results provide vital information that can be used as a guide for biofertilizer application and contribute to the sustainable production of *P*. *ginseng*. This work also offers effective approaches to guarantee the safe medical use of the plant.

## Additional files

**Additional file 1: Table S1.** Barcodes, OTUs and numbers of bacterial sequences in each sample. **Table S2.** Soil chemical properties in the soils of *P. ginseng* plants. **Table S3.** The height and leaf area of *P. ginseng* plants in different treatments. **Table S4.** Calibration curves, linearity, precision, repeatability, stability and recovery rate of six ginsenosides. **Figure S1.** The emergence rate of *P. ginseng* plants in 2015 (A) and 2016 (B). **Figure S2.** Incidence rate of *P. ginseng* damping-off. **Figure S3.** The relative abundance of bacterial taxa at the level of phylum. **Figure S4.** Relative abundance (> 0.10%) of bacterial groups in soils of different treatments during developmental stages of *P. ginseng*.

**Additional file 2.** Minimum Standards of Reporting Checklist.

## Data Availability

The 16S rRNA sequences of endophytes used in this manuscript have been submitted to the NCBI and the Accession number is SRP131253. Most of the data generated of analyzed during the study are included in this article and its Additional file 1.

## Abbreviations

rRNA: ribosomal RNA
PCR: polymerase chain reaction
PCoA: principal coordinates analysis
RDP: ribosomal database project
OTUs: operational taxonomic units
*H*’: Shannon index
PGPR: plant growth-promoting rhizobacteria
