# Supplementary material for: Biofertilizers regulate the soil microbial community and enhance Panax ginseng yields
Source: Chin Med. 2019 May 23;14:20. doi: 10.1186/s13020-019-0241-1 (PMC6533694; doi:10.1186/s13020-019-0241-1)
Supplement: Supplementary file 1 — Additional file 1: Table S1. Barcodes, OTUs and numbers of bacterial sequences in each sample. Table S2. Soil chemical properties in the soils of P. ginseng plants. Table S3. The height and leaf area of P. ginseng plants in different treatments. Table S4. Calibration curves, linearity, precision, repeatability, stability and recovery rate of six ginsenosides. Figure S1. The emergence rate of P. ginseng plants in 2015 (A) and 2016 (B). Figure S2. Incidence rate of P. ginseng damping-off. Figure S3. The relative abundance of bacterial taxa at the level of phylum. Figure S4. Relative abundance (> 0.10%) of bacterial groups in soils of different treatments during developmental stages of P. ginseng. [file 13020_2019_241_MOESM1_ESM.docx]

## Materials and methods

**Analysis of emergence rate of *Panax ginseng*.** The emergence rate *P. ginseng* was calculated by using the numbers of emerging *P. ginseng* seedlings divided by the total numbers of transplanted seedlings in May of 2015 and 2016.

**Analysis of soil chemical properties.** Soil pH was analyzed with a soil: water ratio of 1:2.5 (w/v). Total N and organic contents were measured using Kjeldahl digestion and dichromate oxidation, respectively [1,2]. Available phosphorus (AP) in soil was measured by Mo-Sb anti-spectrophotometry method [3]. Available K was extracted by ammonium acetate and measured by flame photometry.

**Analysis of aboveground growth of *P. ginseng* seedlings.** *P. ginseng* undergoes developmental stages of vegetative, flower, fruit, and root growth each year. The aboveground growth is mainly involved in the stages of vegetative and flower. We analyzed the height and leaf area of *P. ginseng* during vegetative and flowering stages. The leaf area was calculated by leaf length multiplying by leaf width.

## Results

The linearity of all calibration curves were *R*^2^>0.999, and the established HPLC methods could be used for accurate and sensitive quantitative analysis (Table S4).

**Table S1.** Barcodes, OTUs and numbers of bacterial sequences in each sample

| Treatments | Vegetable stage | | | Flowering stage | | | Fruiting stage | | | Root stage | | |
| --- | --- | --- | --- | --- | --- | --- | --- | --- | --- | --- | --- | --- |
|  | Barcodes | OTUs | Clean data | Barcodes | OTUs | Clean data | Barcodes | OTUs | Clean data | Barcodes | OTUs | Clean data |
| CK1 | CGTACTTA | 2913 | 105269 | AGGCGTAT | 2943 | 71295 | TTGCCATG | 2565 | 113651 | ACTGCATA | 2905 | 113588 |
| CK2 | CTAGCCAA | 2631 | 100384 | CAGGTCGA | 2423 | 119231 | TAGCCATA | 2394 | 96779 | AGCTGCGT | 2565 | 121170 |
| CK3 | TCAGGTTA | 2896 | 87230 | CAGCTCTA | 2836 | 136911 | TGCTCAGA | 2585 | 51144 | AGTATCCT | 2705 | 55407 |
| T1-L-1 | ACCTTGCT | 2645 | 30209 | TGCATACA | 2673 | 109150 | CCAGCATA | 2584 | 55310 | ATCGGATA | 2665 | 80017 |
| T1-L-2 | ATCGATAC | 2572 | 69513 | CAGTACTA | 2592 | 85235 | CCTGCATT | 2565 | 31074 | TCACGATC | 2877 | 84791 |
| T1-L-3 | CGTTATAT | 3056 | 55976 | ATGGGATA | 2867 | 50532 | CCGCCTTA | 2913 | 55645 | ACATCTTA | 2708 | 61980 |
| T1-M-1 | ACTGCATA | 2868 | 104301 | CTGCTCAA | 2634 | 91543 | CCAGGACT | 2443 | 78654 | TCGTATTA | 2812 | 87437 |
| T1-M-2 | AGCTGCGT | 2965 | 76692 | ACCCTGAA | 2854 | 47828 | CCGACATT | 2777 | 103062 | AGTCGACA | 2797 | 35784 |
| T1-M-3 | AGTATCCT | 2605 | 80457 | CGATTAGA | 2834 | 116134 | CCGATTTA | 2707 | 64045 | AGTCTATT | 2969 | 93072 |
| T1-H-1 | TGCACCTA | 2550 | 53887 | CGATGTTA | 2580 | 119182 | TCCAGACA | 2496 | 32631 | ACCTTGCT | 2575 | 110144 |
| T1-H-2 | ACGGGATC | 2938 | 75905 | CTAGTAAT | 2959 | 119694 | TGCCGATA | 2837 | 32033 | ATCGATAC | 2773 | 113148 |
| T1-H-3 | AGGAGACT | 2805 | 67639 | CTGACAAT | 2695 | 55616 | TAGAGACA | 2592 | 84789 | CGTTATAT | 2678 | 179886 |
| T2-L-1 | AGTAGACT | 3122 | 85896 | TGCGTATA | 2901 | 32694 | TGCACCTA | 2834 | 201472 | CGATGTTA | 2880 | 32152 |
| T2-L-2 | ATGTTCGT | 3039 | 72311 | TCCGGAAT | 2725 | 53512 | ACGGGATC | 2611 | 31762 | CTAGTAAT | 2662 | 30120 |
| T2-L-3 | CAGTCTGA | 3033 | 31455 | TGGCCATT | 3015 | 84146 | AGGAGACT | 2598 | 17254 | CTGACAAT | 2937 | 45117 |
| T2-M-1 | ATCGGATA | 3016 | 86656 | AGACGATC | 2892 | 68030 | CGTACTTA | 2919 | 24241 | AGGCGTAT | 2927 | 124114 |
| T2-M-2 | TCACGATC | 3020 | 77065 | TACGCTAA | 2950 | 65860 | CTAGCCAA | 2883 | 57776 | CAGGTCGA | 2915 | 126692 |
| T2-M-3 | ACATCTTA | 3097 | 72755 | TCGAGACA | 2946 | 76303 | TCAGGTTA | 2693 | 64992 | CAGCTCTA | 2822 | 33438 |
| T2-H-1 | TCGTATTA | 2818 | 46827 | TGCCTAAA | 2942 | 60345 | CCTCCATA | 2775 | 57813 | AGTAGACT | 2899 | 75190 |
| T2-H-2 | AGTCGACA | 2888 | 15410 | CGATCTTA | 2732 | 16646 | CCATCAGA | 2431 | 63505 | ATGTTCGT | 2675 | 69169 |
| T2-H-3 | AGTCTATT | 2924 | 61207 | CTGGACTA | 2734 | 238940 | CCATGATA | 2727 | 44026 | CAGTCTGA | 2739 | 18968 |

CK represented the treatment without biofertilizers. T1-L, T1-M and T1-H presented treatments with growth-promoting biofertilizers at low, middle, and high concentrations, respectively. T2-L, T2-M, and T2-H presented treatments with disease-biocontrol biofertilizers at low, middle, and high concentrations, respectively. -1, -2 and -3 presented three replicates.

**Table S2.** Soil chemical properties in the soils of *P. ginseng* plants

| Treatments | pH | Total N  (g kg^-1^) | Organic content  (g kg^-1^) | Available P  (mg kg^-1^) | Available K  (mg kg^-1^) |
| --- | --- | --- | --- | --- | --- |
| CK | 5.97±0.33a | 5.26±0.39a | 116.10±5.85a | 47.58±3.64a | 267.14±31.20a |
| T1-L | 5.95±0.24a | 5.29±0.35a | 117.11±6.10a | 45.57±5.79a | 260.46±30.12a |
| T1-M | 5.94±0.26a | 5.32±0.64a | 115.87±6.19a | 46.64±5.14a | 249.89±27.62a |
| T1-H | 5.95±0.41a | 5.49±0.37a | 117.32±12.01a | 44.38±3.67a | 265.25±16.06a |
| T2-L | 5.99±0.43a | 5.34±0.26a | 120.42±12.11a | 48.64±5.78a | 257.78±28.24a |
| T2-M | 5.92±0.35a | 5.37±0.29a | 126.33±6.18a | 48.16±6.94a | 267.18±26.17a |
| T2-H | 5.91±0.23a | 5.24±0.64a | 115.67±6.78a | 47.67±9.10a | 245.97±33.09a |
| T3-L | 5.97±0.33a | 5.52±0.57a | 121.18±8.49a | 49.16±5.78a | 266.12±29.37a |
| T3-M | 5.96±0.27a | 5.51±0.62a | 126.48±9.87a | 45.28±5.69a | 263.17±27.16a |
| T3-H | 5.91±0.22a | 5.47±0.41a | 118.75±6.21a | 47.92±4.67a | 259.18±23.55a |

Data were presented as the mean ± SD of n = 3, and same letter indicated non-significant differences in the soil chemical characteristics of different treatments at the level of 0.05.

**Table S3.** The height and leaf area of *P. ginseng* plants in different treatments.

| Date | Treatments | Vegetable stage | | Flowering stage | |
| --- | --- | --- | --- | --- | --- |
|  |  | Height (cm) | Leaf area (cm^2^) | Height (cm) | Leaf area (cm^2^) |
| 2015 | CK | 10.26±0.26a | 26.12±2.44a | 11.85±1.33a | 32.00 ±2.16a |
|  | T1-L | 9.62±0.55a | 25.66±2.16a | 10.66±1.07a | 31.66±3.11a |
|  | T1-M | 9.53±0.49a | 25.49±2.18a | 10.18±0.95a | 31.56±3.16a |
|  | T1-H | 10.12±0.68a | 26.77±1.59a | 11.56±0.85a | 41.75±2.59a |
|  | T2-L | 10.86±0.51a | 27.49±1.77a | 12.26±0.77a | 32.26±3.12a |
|  | T2-M | 10.79±0.46a | 28.66±2.16a | 12.02±1.24a | 32.16±1.56a |
|  | T2-H | 10.32±1.32a | 26.11±2.88a | 11.89±1.34a | 33.35±4.66a |
|  | T3-L | 10.85±0.26a | 26.55±2.49a | 12.31±1.67a | 33.12±2.67a |
|  | T3-M | 11.26±0.45a | 27.88±2.17a | 12.58±1.36a | 35.22±3.18a |
|  | T3-H | 10.11±0.35a | 26.18±3.67a | 11.89±0.99a | 35.19±4.66a |
| Date | Treatments | Vegetable stage | | Flowering stage | |
|  |  | Height (cm) | Leaf area (cm^2^) | Height (cm) | Leaf area (cm^2^) |
| 2016 | CK | 21.20±1.77a | 51.92±3.22a | 22.27±3.27a | 56.32±8.32a |
|  | T1-L | 19.79±2.68a | 40.59±3.16a | 20.17±4.12a | 55.49±6.99a |
|  | T1-M | 19.77±2.78a | 39.39±1.55a | 19.89±3.11a | 52.10±4.99a |
|  | T1-H | 21.89±2.04a | 46.04±2.75a | 22.69±3.61a | 64.90±6.81a |
|  | T2-L | 19.78±3.11a | 36.35±1.69a | 18.80±7.61a | 54.36±8.32a |
|  | T2-M | 22.19±1.79a | 39.06±4.16a | 20.16±4.60a | 57.28±6.18a |
|  | T2-H | 20.89±0.99a | 44.31±3.11a | 20.66±3.16a | 63.12±7.98a |
|  | T3-L | 18.77±1.22a | 46.30±4.17a | 20.35±4.30a | 59.35±7.11a |
|  | T3-M | 22.67±4.31a | 48.29±1.29a | 23.94±4.41a | 64.29±9.22a |
|  | T3-H | 21.98±2.67a | 47.87±3.66a | 22.27±5.03a | 67.11±8.14a |

CK represented the treatment without biofertilizers. T1-L, T1-M and T1-H presented treatments with growth-promoting biofertilizers at low, middle, and high concentrations, respectively. T2-L, T2-M, and T2-H presented treatments with disease-biocontrol biofertilizers at low, middle, and high concentrations, respectively. Data were presented as the mean ± SD of n = 3, and same letter indicated non-significant differences among treatments at the level of 0.05.

**Table S4.** Calibration curves, linearity, precision, repeatability, stability and recovery rate of six ginsenosides.

| Ginsenosides | Calibration curves | *R*^2^ | Linearity  (ug) | RSD% (*n*=6) | | | Recovery rate (%) (RSD%) |
| --- | --- | --- | --- | --- | --- | --- | --- |
|  |  |  |  | Precision | Repeatability | Stability |  |
| Rg1 | Y=102.17X+23.16 | 0.9992 | 0.099-10.12 | 0.12 | 1.55 | 2.11 | 96.99(2.01) |
| Re | Y=232.27X+22.36 | 0.9997 | 0.097-1.99 | 0.06 | 0.97 | 2.67 | 97.22(1.87) |
| Rb1 | Y=292.36X+3.49 | 0.9993 | 0.096-1.96 | 0.07 | 0.78 | 2.33 | 101.34(2.18) |
| Rc | Y=192.46X+2.87 | 0.9995 | 0.099-1.99 | 0.12 | 0.41 | 2.98 | 102.17(1.26) |
| Rb2 | Y=233.19X+3.27 | 0.9992 | 0.092-1.93 | 0.33 | 1.76 | 2.66 | 98.89(2.66) |
| Rd | Y=299.86X+2.47 | 0.9998 | 0.097-1.99 | 1.21 | 0.92 | 2.16 | 100.16(1.66) |

**Figure S1**


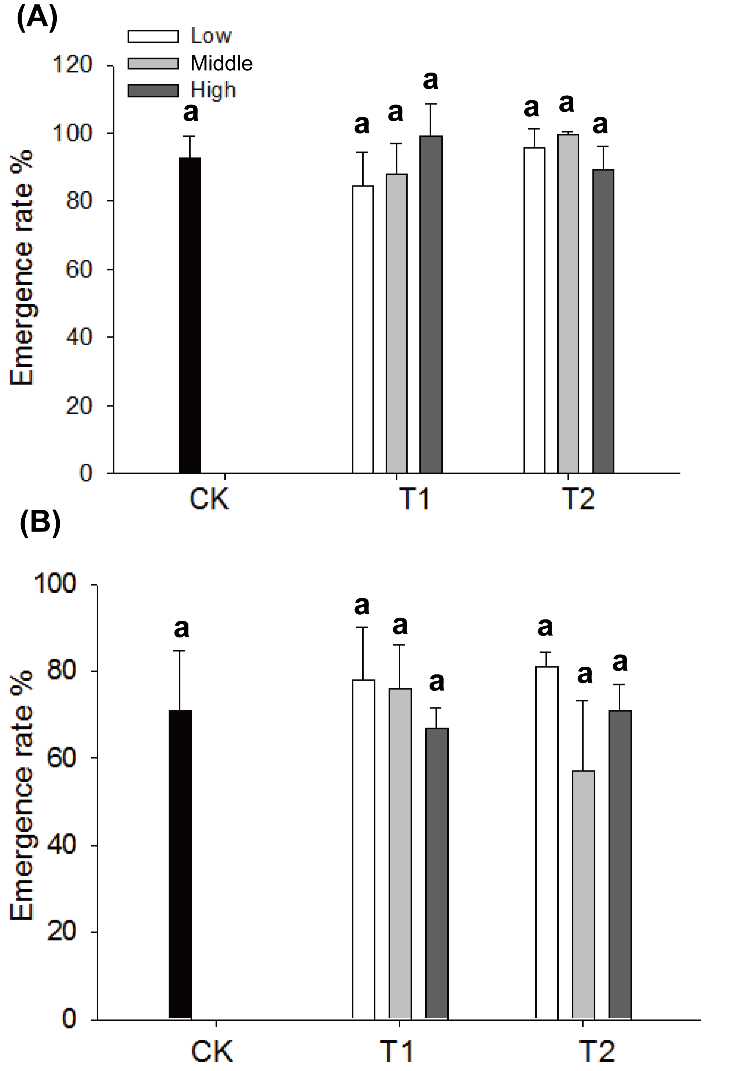


**Figure S1.** The emergence rate of *P. ginseng* plants in 2015 (A) and 2016 (B). CK represented the treatment without biofertilizers. T1 and T2 presented treatments with growth-promoting and disease-biocontrol biofertilizers, respectively. Data were presented as mean (*n*=3) ± SD. Identical letters denoted non-significant difference among treatments at 0.05 level.

**Figure S2**


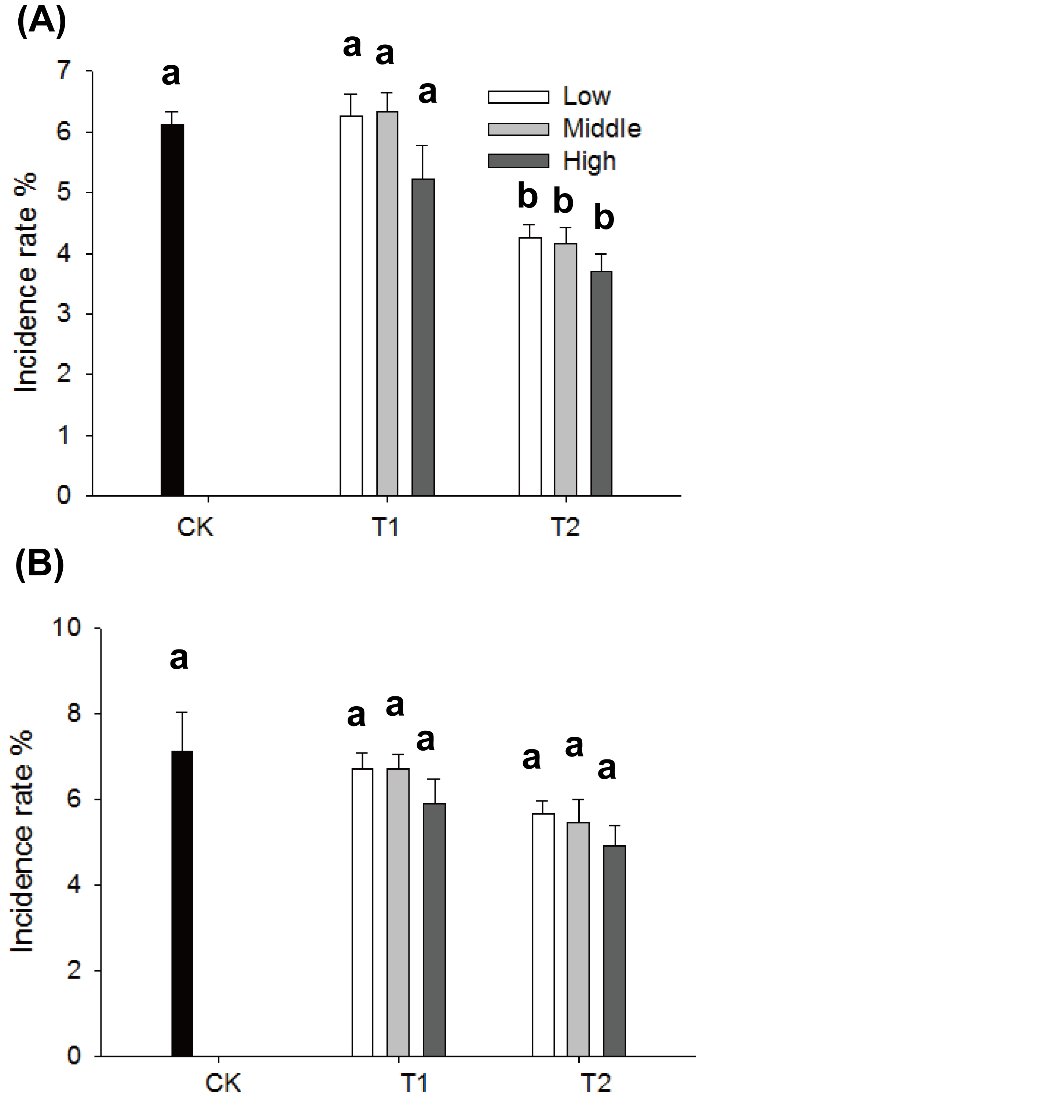


**Figure S2.** Incidence rate of *P. ginseng* damping-off. (A) Incidence rate of damping-off in 2015. (B) Incidence rate of damping-off in 2016. CK represents the treatment without biofertilizers. T1 and T2 present treatments with growth-promoting and disease-biocontrol biofertilizers, respectively. Data are presented as mean (*n*=3) ± SD. Identical letters denote non-significant difference among treatments at 0.05 level.

**Figure S3**


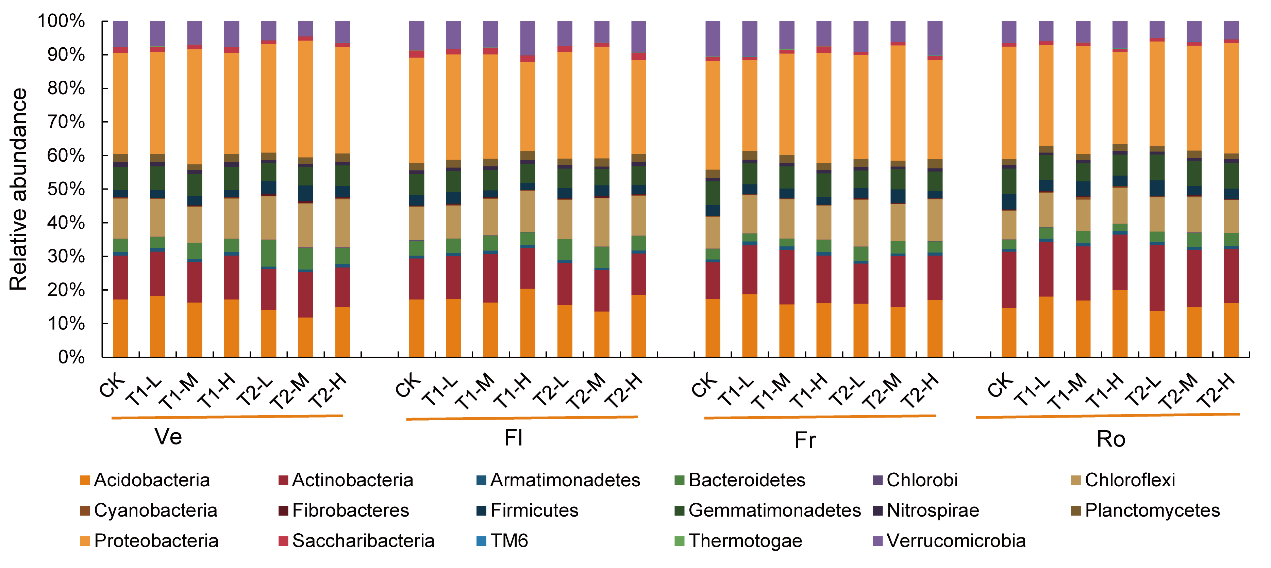


**Figure S3.** The relative abundance of bacterial taxa at the level of phylum. T1-L, T1-M and T1-H presented treatments with growth-promoting biofertilizers at low, middle, and high concentrations, respectively. T2-L, T2-M, and T2-H presented treatments with disease-biocontrol biofertilizers at low, middle, and high concentrations, respectively. Ve, Fl, Fr and Ro represented the stages of vegetative, flower, fruit and root growth. Data were presented as mean (*n*=3).

**Figure S4**


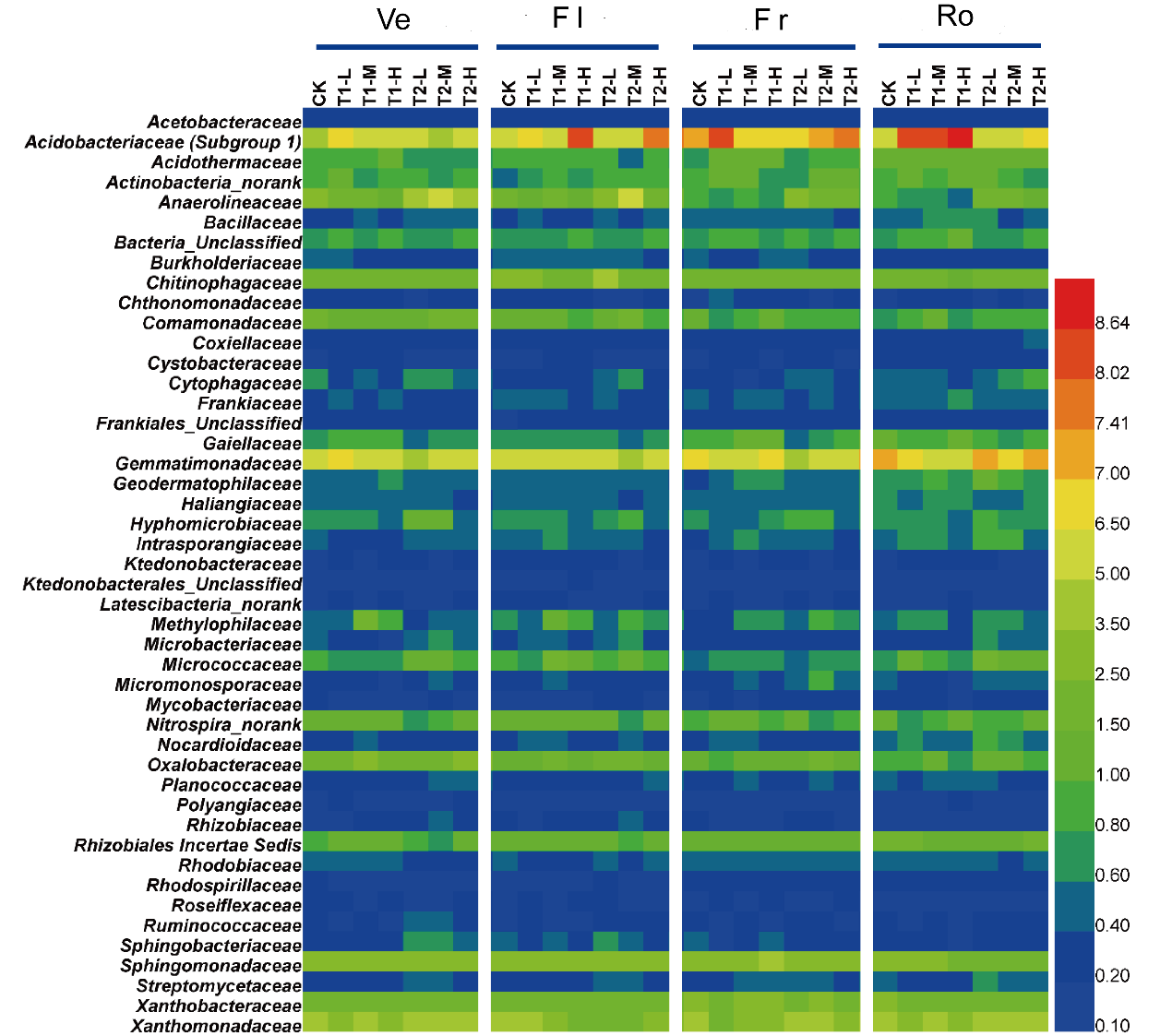


**Figure S4.** Relative abundance (>0.10%) of bacterial groups in soils of different treatments during developmental stages of *P. ginseng*. Ve, Fl, Fr and Ro represented the stages of vegetative, flower, fruit and root growth. T1-L, T1-M and T1-H presented treatments with growth-promoting biofertilizers at low, middle, and high concentrations, respectively. T2-L, T2-M, and T2-H presented treatments with disease-biocontrol biofertilizers at low, middle, and high concentrations, respectively. Data were presented as mean (*n*=3).

## References

[1] Keeney DR, Nelson DW. Nitrogen-inorganic forms. Methods of soil analysis. Part 2: chemical and microbiological properties. Madison: American Society of Agronomy 1982; pp 595.

[2] Mebius LJA. A rapid method for the determination of organic carbon in soil. Analytica Chimica Acta 1960; 22*:* 120-124.

[3] Olsen SR, Cole CV, Watanabe FS, Dean LA. Estimation of available phosphorus in soils by extraction with sodium bicarbonate. United States Department of Agriculture in cooperation with the Colorado Agricultural Experiment Station 1954; pp 939.
